# Supplementary material for: The Effects of a Mediterranean Diet on Metabolic Hormones and Cytokines in Amyotrophic Lateral Sclerosis Patients: A Prospective Interventional Study
Source: Nutrients. 2025 Apr 25;17(9):1437. doi: 10.3390/nu17091437 (PMC12073196; doi:10.3390/nu17091437)
Supplement: Supplementary file 1 [file nutrients-17-01437-s001.zip › nutrients-3572325-supplementary.pdf]

Table S1. Differences in serum values of metabolic hormones at the 3 visits. Values of metabolic biomarkers were shown as mean±standard deviation.

Variable	T0 (n=44)	T1 (n=36)	T2 (n=30)	T0 vs T1	T1 vs T2	T0 vs T2
Amylin Total	282.14±266.81	228.77±204.29	235.61±206.67	0.378	0.907	0.483
C-peptide	3813.60±1966.65	3171.03±2013.90	9532.28±4000.35	0.154	<0.001*	<0.001*
Active Ghrelin	30.45±17.45	29.84±23.37	24.5±18.67	0.895	0.355	0.196
GIP	6066.00±4394.22	6586.53±4557.95	6002.17±5290.31	0.606	0.631	0.955
GLP-1	118.1±76.3	115.96±75.61	59.89±57.45	0.902	0.002*	0.001*
Glucagon	79.84±49.10	77.42±49.81	56.92±48.22	0.828	0.096	0.051
Insulin	480.34±369.33	439.38±405.1	214.15±213.25	0.692	0.042*	0.009*
Leptin	4956.31±3993.75	3196.34±2807.09	4935.22±4778.7	0.038*	0.089	0.984
MCP-1	4685.07±2233.92	6174.59±2623.5	6083.55±2047.08	0.009*	0.879	0.008*
PP	4894.93±4725.08	5598.81±4197.55	2422.45±2114.13	0.498	0.003*	0.034*
PYY	110.86±95.32	102.93±72.66	95.15±82.60	0.682	0.685	0.465

Table S2. Differences in serum values of cytokines at the 3 visits. Abbreviation: IFNγ (interferon gamma), CCL20 (chemokine C-C motif ligand 20), GM-CSF (granulocyte-macrophage colony-stimulating factor), interleukin (IL)-1β, IL-2, IL-4, IL-5, IL-6, IL-9, IL-10, IL-12P70, IL-13, IL-15, IL-17A, IL17E/IL-25, IL-17F, IL-21, IL-22, IL-23, IL-27, IL-28A, IL-31, IL-33, TNFα (tumor necrosis factor alpha), TNFβ.

Variable	T0 (n=44)	T1 (n=36)	T2 (n=30)	T0 vs T1	T1 vs T2	T0 vs T2
IFNγ	14.4±4.47	13.58±4.77	12.62±5.49	0.434	0.447	0.129
IL-10	31.01±19.29	35.81±31.75	30.8±24.09	0.412	0.480	0.967
CCL20	47.48±38.03	49.09±46.63	52.72±39.18	0.868	0.741	0.567
IL-12P70	20.6±7.03	18.6±4.73	16.32±4.99	0.148	0.062	0.005*
IL-13	77.4±63.06	79.03±78.94	48.78±33.91	0.918	0.055	0.026*
IL-15	41.86±9.66	41.22±18.22	38.77±11.48	0.841	0.525	0.214
IL-17A	22.99±13.40	19.17±3.83	26.00±7.23	0.102	<0.001*	0.265
IL-22	64.73±47.33	64.68±53.87	38.63±20.01	0.996	0.020*	0.009*
IL-9	24.99±9.55	23.44±7.49	21.2±4.53	0.431	0.156	0.047*
IL-1b	30.64±19.56	26.81±17.69	28.72±14.97	0.366	0.641	0.651
IL-33	32.36±25.92	30.83±28.07	31.2±21.46	0.810	0.955	0.848
IL-2	31.40±17.24	28.42±14.56	20.30±13.15	0.412	0.022*	0.004*
IL-21	21.52±9.25	19.71±7.84	21.08±5.80	0.353	0.429	0.818
IL-4	25.9±19.9	21.3±17.34	30.56±19.12	0.302	0.057	0.349
IL-23	29.24±24.26	30.28±28.71	23.07±21.19	0.866	0.281	0.288
IL-5	21.53±11.31	19.32±11.56	21.38±9.94	0.391	0.445	0.953
IL-6	36.63±23.76	36.77±31.16	48.15±39.07	0.983	0.214	0.135
IL-17E/IL-25	36.43±31.28	32.44±27.16	30.2±26.08	0.567	0.748	0.397
IL-27	201.1±75.84	187.82±77.3	211.75±58.83	0.457	0.183	0.534

IL-31	37±26.63	37.97±32.1	31.24±26.58	0.887	0.384	0.388
TNFα	75.90±52.40	62.31±38.29	94.60±42.02	0.198	0.002*	0.108
TNFß	24.89±15.74	20.89±9.3	18.54±7.1	0.204	0.297	0.063
IL-28A	23.12±10.05	22.29±15.86	23.1±14.26	0.789	0.841	0.996
IL-17F	57.18±47.03	49.75±32.04	43.96±39.22	0.436	0.525	0.225
GM-CSF	20.98±16.12	18.54±12.55	21.53±13.45	0.461	0.354	0.877
